# Elucidation of mechanisms of actions of thymoquinone-enriched methanolic and volatile oil extracts from *Nigella sativa* against cardiovascular risk parameters in experimental hyperlipidemia

**DOI:** 10.1186/1476-511X-12-86

**Published:** 2013-06-13

**Authors:** Shafeeque Ahmad, Zafarul H Beg

**Affiliations:** 1 Department of Biochemistry, Jawahar Lal Nehru Medical College, Aligarh Muslim University, Aligarh, UP 202 002, India

**Keywords:** *Nigella sativa*, Methanolic extract, Volatile oil, GC-MS, HMG-CoA reductase, Lipoprotein oxidation

## Abstract

**Background:**

*Nigella sativa* belonging to the Ranunculaceae family has been reported to use for thousands of years as protective and curative traditional medicine against a number of diseases. GC-MS analysis of methanolic extract (ME) and volatile oil (VO) extracted from *Nigella sativa* seed oil was performed by two different mass spectrometry libraries, WIlEY8 and NIST05s. The cholesterol lowering and antioxidant actions of VO and ME fractions were investigated in atherogenic suspension fed rats.

**Methods:**

In this study, four groups of male Wistar rats were used: normolipidemic control (NLP-C), hyperlipidemic control (HLP-C), methanolic extract (HLP-ME) and volatile oil treated (HLP-VO) groups for 30 days of duration. P value < 0.05 was assumed as significant data in groups.

**Results:**

Administration of atherogenic suspension to male Wistar rats for 30 days resulted in a marked increase of plasma triglycerides and total cholesterol, and significant change in plasma lipoprotein levels along with a decrease in antioxidant arylesterase activity in hyperlipidemic control (HLP-C) group. The oral feeding of 100 mg ME or 20 mg VO per rat/day effectively reduced the plasma triglycerides to near normal level, while high density lipoprotein cholesterol and its subfraction along with arylesterase activity levels were significantly increased. The test fractions elicited a significant decrease in hepatic HMG-CoA reductase activity. The fractions significantly blocked the *ex vivo* basal and *in vitro* maximal formation of conjugated diene and malondialdehyde, and lengthened the lag times of low density lipoprotein, small dense low density lipoprotein and large buoyant low density lipoprotein. ME possessing ω-6 linoleic acid along with palmitic acid active compounds was more effective than VO extract containing thymol and isothymol phenolic antioxidant compounds, thymoquinone phenolic compound common to the both extracts, via reduction in hepatic HMG-CoA reductase activity as well as antioxidant mechanisms.

**Conclusion:**

The both extracts especially, ME significantly improve cardiovascular risk parameters in treated rats, and can be used in reactive oxygen species disorders such as cardiovascular diseases.

## Background

The World Health Organization (WHO) has declared that due to cardiovascular disease (CVD), the death toll of people will reach approximately 23.6 million around the world by 2030 [1]. It is well known that hypercholesterolemic patients are more prone to heart attack than normal blood lipid profiles. According to the WHO report, consumption of unhealthy diets, rich in fat, salt and free sugar, and low in complex carbohydrates, fruits and vegetables are responsible for CVD [2]. Diets rich in cholesterol produce free radicals, followed by oxidative stress and hypercholesterolemia [3,4]. Oxidative stress is one of the factors that causes rise in the level of blood cholesterol and it may result in atherogenesis [5]. High concentration of LDL-C in plasma is one of the major causes of coronary heart disease (CHD) as evidenced by epidemiological studies [6]. In comparison to lb-LDL, sd-LDL is highly atherogenic because of the presence of properties like enhanced susceptibility to oxidation, higher penetration in the arterial wall, lower binding affinity to the LDL-receptor, and prolonged plasma half life [7,8]. Hence, it will be focusing attention on sd-LDL as a new precise and useful CHD marker [9-11]. Blood cholesterol is recommended to be within normal range to preserve normal cell functions. At present, there are several methods known to control blood cholesterol levels, including dietary fats, bile acids sequestering agents and use of HMG-CoA reductase inhibitors. Among them, HMG-CoA reductase is the key enzyme in the cholesterol biosynthesis pathway that could be an important target of an inhibitor for managing blood cholesterol levels [12].

Use of the modern drugs explores various drawbacks that are expensive, beyond the reach of a majority of the population of the world and multiple side effects. Therefore, today drugs from indigenous sources should be designed and suitable different combinations of such drugs tested for therapeutic purposes, which posses minimal adverse effects and multiple targets in preventing and curing diseases. A number of epidemiological studies have been reported that plant foods carry a high quantity of phenolic compounds, are effective against fatal chronic diseases like cancer, neurodegenerative and CVD [13-15]. Such compounds show antioxidant properties. Their free radical scavenging properties solely rely upon constituents as well as synergistic interactions [16]. Various types of reactive oxygen species (ROS) are generated in our body and their removal must be continually done for the maintenance of normal cellular functions. Phenolic compounds (POH) deactivate various types of free radicals such as hydroxyl radicals and peroxyl radicals (R^•^) by donating hydrogen, which interrupts chain reactions:

$$\begin{array}{l} R^{•}+\text{POH}\to {\mathrm{R-H}+\mathrm{PO}}^{•}\left(\text{Interruption}\;\text{reaction}\right)\\ {\mathrm{PO}}^{•}{+\mathrm{PO}}^{•}\to \mathrm{PO-OP}\;\left(\text{Stabilization}\;\text{reaction}\right) \end{array}$$

In the above reactions, stabilization of phenoxyl radicals (PO^•^) is possible through resonance and/or intramolecular hydrogen bonding [17]. Thus, consumption of phenolic compounds may be useful to the health. Recently, *Nigella sativa* (NS) has been reported to possess multiple health beneficial properties. Several studies have been reported that methanolic extract (ME) [14], volatile oil (VO) [18] from black cumin and their principal active constituent thymoquinone [19], show antioxidant properties. Phenolic compounds such as thymol, isothymol have been shown to have antioxidant actions [20], and essential fatty acid, ω-6 linoleic acid [21] or ω-6 linoleic acid with palmitic acid have hypolipidemic property [22].

Elevated levels of TG and TG rich VLDL, these two parameters are regarded as risk factors for atherosclerosis [23,24], while an elevated level of plasma HDL-C is considered beneficial to the health because these are inversely correlated with the risk factor for atherosclerosis [25]. In agreement with these concepts, drugs that cause reduction of TG, TG rich VLDL and increase in HDL would be helpful in decrease in the morbidity and mortality from CAD [25,26]. HDL is assumed a key lipoprotein that helps lipid removal from macrophage foam cells in the arterial wall, and thus protects LDL oxidation. Several studies have been performed using albino Wistar rats as experimental atherosclerosis model, and they found it suitable and effective model to be used in condition of hypercholesterolemia and atherosclerosis [27,28]. In the present study, we have performed gas chromatography (GC) analysis of the ME and VO, fractionated from NS seed oil and identified their constituents by using two different mass spectrometry (MS) libraries. In order to understand the mechanisms of lipid lowering actions of these test fractions, we explored the hepatic HMG-CoA reductase activity as well as their protective effect on HDL-linked arylesterase activity, in rats for 30 days. Further, the antioxidant protection of the test fractions afforded to LDL and its density subfractions, sd-LDL and lb-LDL, from *in vivo* and Cu^2+^-catalyzed *in vitro* oxidation was investigated. This objective was achieved by measuring *ex vivo* basal and *in vitro* maximal CD formations and lag times, as well as *ex vivo* basal and maximal production of MDA in LDL, sd-LDL and lb-LDL lipoproteins isolated from plasma of experimental animals.

## Materials and methods

### Materials

Export quality, edible *Nigella sativa* (NS) seed oil, was bought from a local store. Phenyl acetate was procured from Sigma-Aldrich Inc. USA. Highly purified thiobarbituric acid and hydroxylamine hydrochloride were purchased from HiMedia Laboratories Pvt. Ltd. (Mumbai, India), while phosphotungstic acid was bought from Loba Chemie (Mumbai, India). And HMG-CoA was procured from Pharmacia, USA. Mevalonolactone was purchased from United States Bioch. Corp., USA, while sodium dodecyl sulfate was obtained from Bio-Rad Laboratories USA. Triglycerides assay kit for determination of triglycerides was procured from Autospan Kit SPAN Diagnostics (New Delhi, India). Other chemicals and reagents were used in this research of analytical grade.

### Preparation of methanolic extract (ME) and volatile oil (VO) extracts from NS seed oil

The ME fraction, used in the present study, was extracted by stirring 10 g of the NS seed oil with 100 ml of methanol for 100 min at ambient temperature. The methanol layer was removed, and then evaporated at 45°C. The VO fraction was extracted from NS seed oil by steam distillation. The procedure is essentially the same as described for the isolation of VO extract from NS seeds [29] with minor modification. Briefly, 400 ml of distilled water was added to a distillation flask containing 12 g of NS seed oil, and the temperature was set to the boiling point. The process of distillation was continued until about 150 ml of the distillate containing VO was collected in a dark glass bottle. The distillate was extracted with 50 ml of diethyl ether, moisture was removed by the addition of anhydrous sodium sulphate and the resultant ether extract was evaporated at 40°C. The ME and VO extracts thus obtained were stored under nitrogen in dark colored bottles at 4°C.

### GC-MS analysis

GC-MS analyses of ME and VO extracted from NS oil were performed by GCMS (QP2010 plus system Shimadzu). Separation was carried out at chromatograph column of SLB60m × ID 0.25 mm × thickness 0.25 μm. Column oven temperature was initially set at 70°C for 2min, which was increased to 310°C at the rate of 5°C per min with 20 min hold. The carrier gas, helium was used with a flow of 1.21 ml per min. The split ratio was adjusted to 30.0. Injector temperature was adjusted at 270°C. Ionization voltage was 70 eV. The constituents present in the samples were identified by matching their mass spectra with two mass spectra libraries, namely WIlEY8.LIB and NIST05s.LIB. The software of the system determined relative percentage amount of the compounds that exist in the both samples.

### Animals and treatments

The Board of Studies of the Biochemistry Department and the Animal Ethics Committee of J.N. Medical College, A.M.U., Aligarh, where the present research work was performed, approved the experimental study. Healthy male Wistar albino rats, weighting 180–210 g from inbred colony maintained by central animal facility of J.N. Medical College, were allowed free access to standard rat chow and water. Ten percent ME and 2% VO suspensions were prepared by dissolving in DMSO (12.5%) and then homogenized with saline. The rats were divided into the following groups:

Normolipidemic control (NLP-C): This group of animals containing five rats was orally administered 1 ml solution of saline containing 12.5% DMSO, twice 0.5 ml each in the morning and evening per rat per day for 30 days of duration.

Hyperlipidemic control (HLP-C): For the induction of hyperlipidemia, before the administration of 1.0 ml atherogenic suspension, this experimental group received 1.0 ml of saline containing 12.5% DMSO, twice 0.5 ml each in the morning and evening per rat per day for 30 days.

Hyperlipidemic methanolic extract (HLP-ME): One milliliter of 10% ME suspension was given to the rats, before the administration of 1.0 ml of atherogenic suspension, twice 0.5 ml each in the morning and evening per rat per day for 30 days.

Hyperlipidemic volatile oil (HLP-VO): One milliliter of 2% VO suspension was administered, before the administration of 1.0 ml of atherogenic suspension, twice 0.5 ml each in the morning and evening per rat per day for 30 days.

As in previous studies, ME and VO were taken at dose of 810 mg and 400 mg/day/kg body weight of male Wistar rats, respectively [30,31]. The LD_50_ of ME was not found at four different doses, 6, 9, 14 and 21 g/kg, and further no hepatic toxicity of ME at 6 g/day/kg body weight of mice was reported [32], and the LD_50_ of VO was found to be 0.5 ml/day/kg body weight of male albino mice [33]. So based on these reports, we took single, safe and effective dose of ME and VO.

### Plasma separation from blood

At the end of the experiment, rats were anaesthetized and blood was taken out by cardiac puncture from overnight fasted rats in each group. The blood was collected in heparinized tubes. After incubation at 4°C for 2–3 h, blood samples were centrifuged at 2,500 rpm for 30 min. Plasma was aliquoted and stored at 4°C for future use.

### Low density lipoprotein (LDL), small dense (sd)-LDL and large buoyant (lb)-LDL fractionation

Basically the precipitation method for plasma LDL isolation as described by Wieland and Seidel [34] was employed. Briefly, the precipitation buffer consisted of 64 mM trisodium citrate, pH 5.05, plus 50,000 IU/L heparin. Prior to LDL precipitation, plasma samples and precipitation reagent were allowed to equilibrate to room temperature. Then 1.0 ml of plasma was added to 7.0 ml of heparin-citrate buffer, mixed properly, and then it was allowed to stand for 10 min at 22°C. Then it was centrifuged at 1,500 rpm for 10 min at 22°C. The pellet thus obtained was resuspended in 1.0 ml of 0.1 M sodium phosphate buffer saline, pH 7.4. Hirano *et al.*[10] described the isolation of small dense (sd)-LDL and large buoyant (lb)-LDL from isolated LDL which is based on the two-step procedure quantifying sd-LDL from serum by heparin-Mg^2+^ precipitation. In our modified method for the isolation of sd-LDL and lb-LDL from plasma LDL, 0.1 ml precipitation reagent solution prepared by taking 15 IU of heparin and 90 mM MgCl_2_ was added to 0.1 ml of LDL. The sample was properly mixed, then incubated at 37°C for 10 min. Each sample was then incubated in an ice-bath for 15 min. Finally, each sample was centrifuged at 10,000 rpm for 15 min at 4°C. The upper supernatant and lower portions of the samples containing sd-LDL and lb-LDL, respectively were carefully removed and saved. The lb-LDL fraction as pellet was dissolved in 0.1 M sodium phosphate buffer saline, pH 7.4. Appropriate quantities of LDL, sd-LDL and lb-LDL fractions were taken out for further analysis.

### Isolation of plasma very low density lipoprotein (VLDL)

Plasma VLDL was isolated as described by Bachorik and Albers [35]. Briefly, for the isolation of VLDL, 0.075 volume of 10% sodium dodecyl sulfate solution was added to 1 volume of plasma, and then properly mixed. Afterwards, it was incubated for 2 h at 37°C and finally centrifuged at 10,000 rpm for 10 minutes at room temperature. At the top of the centrifuge tube, VLDL pellicle was appeared. The supernatant was carefully removed. The residue of VLDL pellicle was dissolved in one volume of 1% sodium dodecyl sulfate by warming at 37°C.

### Isolation of plasma high density lipoprotein cholesterol (HDL-C) and HDL_3_-C subfraction

High density lipoprotein cholesterol from plasma was isolated by the method of Kostner [36] and HDL_3_-C subfraction of HDL-C was isolated by the procedure as described by Bachorik and Albers [35]. In brief, 0.2 volume of phosphotungstic acid and magnesium chloride (1:1) was added to 1 volume of plasma, and then instantly and properly mixed. It was finally centrifuged for 10 min at 12,000 rpm at room temperature. The clear supernatant was carefully removed and thus represented the plasma HDL-C. For HDL-C fractionation, reagent ‘X’ was prepared by mixing 1 volume of 40 g/L dextran sulfate, Ph 7.0 with 3 volumes of 2M MgCl_2_.6H_2_O, Ph 7.0. To fractionate HDL-C, 0.1 volume of reagent ‘X’ was added to one volume of HDL-C and mixed immediately. Then it was centrifuged at 5,000 rpm for 30 min at 4°C. Aliquots of clear supernatant were used for HDL_3_-C analysis. HDL_2_-C was then calculated as the difference between HDL-C and HDL_3_-C concentration.

### Determination of plasma and lipoprotein cholesterols

Plasma total cholesterol, VLDL, LDL and HDL lipoprotein cholesterols and their fraction cholesterols were determined as previously stated by Ahmad and Beg [37] in detail.

### Determination of plasma triglycerides

Triglycerides present in plasma were determined by enzymatic kit, based on a modified Trinder color reaction, which produces fast, linear, and end point reaction [38]. The intensity of the color produced of the triglycerides in the plasma samples was measured at 500 nm in a Beckman DU 640 spectrophotometer. Triolein was used as standard to calculate triglycerides concentration in plasma samples.

### Determination of hepatic HMG-CoA reductase activity

HMG-CoA reductase enzyme activity in liver homogenate was assayed indirectly by employing the method of Rao and Ramakrishnan [39]. Briefly, fresh 10 g liver was homogenized in 90 ml of saline arsenate. Then equal volume of 10% liver homogenate and 5% perchloric acid (5 ml each) was taken out and thoroughly mixed, and kept for 5 min at room temperature. Afterwards, it was centrifuged at 2,000 rpm for 10 min. Half milliliter of freshly prepared 1 M aqueous hydroxylamine hydrochloride was mixed with one ml of the supernatant obtained after centrifugation. For determination of HMG-CoA reductase, 0.5 ml of alkaline hydroxylamine hydrochloride was added and properly mixed. Then it was allowed to incubate for 5 min at room temperature. Afterwards, 1.5 ml of 0.616 M ferric chloride solution consisted of 5.2% trichloroacetic acid prepared in 0.65 N HCl was added to the above sample and thoroughly mixed, and incubated for 10 min at room temperature. Absorbance was recorded at 540 nm against a reagent blank in a Beckman DU 640 spectrophotometer.

### Determination of plasma arylesterase antioxidant enzyme activity

Plasma arylesterase antioxidant enzyme activity was essentially assayed by the method of Ayub *et al.*[40]. Phenyl acetate was used as the substrate. The reaction mixture consisted of 100 mM tris buffer, pH 8.0, 1 mM CaCl_2_, 1 mM phenyl acetate and suitable aliquots of plasma sample. After 5 min of preincubation period of the samples, the reaction was started by adding the phenyl acetate and incubated for different time intervals at 25°C. Absorbance of initial rates of hydrolysis was determined spectrophotometrically at 270 nm against a reagent blank, while nonenzymatic hydrolysis of phenyl acetate was subtracted from the total rate of hydrolysis. The molar extinction coefficient of 1.31 × 10^3^ M^-1^ cm^-1^ was used to calculate the arylesterase enzyme activity in the plasma sample [41].

### Determination of *ex vivo* and *in vitro* Cu^2+^-mediated susceptibility of LDL, sd-LDL and lb-LDL to oxidation

The *ex vivo* and *in vitro* Cu^2+^-mediated susceptibility of LDL, sd-LDL and lb-LDL to oxidation was determined by as described by Esterbauer *et al.*[42] and Esterbauer *et al.*[43]. Before oxidation studies of lipoprotein fractions by determining the lag phase of conjugated diene formation were dialyzed against 10 mM phosphate buffer saline (PBS), pH 7.4, for 12 h. At different time of interval of oxidation, 100 μg of LDL, sd-LDL or lb-LDL dissolved in 1.0 ml of PBS was added to 0.5 mM EDTA, and then mixed, afterward stored at 4°C and used for the assessment of conjugated diene formation. In another set of zero time *ex vivo* oxidation, one ml aliquot of each sample was added to a tube carrying 0.5 mM EDTA, pH 7.4, for the determination of levels of conjugated diene formation in the lipoprotein isolated from plasma. Then, lipoprotein fractions, LDL, sd-LDL and lb-LDL from plasma were added to CuSO_4_ making final concentration of 5 μM and incubated at 37°C. Durations of 2 h for LDL and lb-LDL, and 30 min for sd-LDL were taken for their oxidation. The conjugated diene formation in plasma lipoproteins was quantified by recording absorbance at 234 nm in a Beckman DU 640 spectrophotometer. Molar extinction coefficient of 2.52 × 10^4^ M^-1^ cm^-1^ was used to calculate conjugated diene formation, and expressed as nmole malondialdehyde (MDA) equivalent per mg LDL, sd-LDL or lb-LDL protein.

Niehaus and Samuelsson [44] described a method to determine MDA content in LDL, sd-LDL and lb-LDL isolated from plasma. In Brief, 2 ml of trichloroacetic acid (15%)-thiobarbituric acid (0.375%)-hydrochloric acid (0.25 N) reagent was added to 0.3 ml of LDL, sd-LDL and lb-LDL, and mixed properly. The solution was kept in a boiling water bath for 15 min. Then flocculent precipitate was removed by centrifugation at 1000 rpm for 10 min. The absorbance of the samples was monitored at 535 nm against a sample blank. The MDA concentration of the lipoprotein samples was determine by using a molar extinction coefficient (1.56 × 10^5^ M^-1^ cm^-1^).

### Protein estimation

The method of Bradford [45] was used for protein estimation of plasma lipoproteins and liver using bovine serum albumin as standard. Suitable aliquots of the samples were precipitated with 10% trichloroacetic acid. The obtained protein pellets were dissolved in 0.5 N NaOH, then suitable portions of the samples were used for protein quantification.

### Statistical analysis

One way analysis of variance (ANOVA) was employed for statistical analysis of the data, then Tukey’s Kramer multiple comparison test (GraphPad software Inc., San Diego, CA, USA) was used. P value < 0.05 was assumed as significant data.

## Results

### GC-MS analysis

Thirty compounds in methanolic extract (ME) were identified by GC-MS analysis and listed in Table 1. As shown in Table 1, the compounds that were examined in ME, consists of a mixture of terpenes, saturated and unsaturated fatty acids along with certain ester form of compounds. The major compounds were limonene (1.13%), butylated hydroxytoluene (2.44%), thymoquinone (2.64%), palmitic acid (2.77%), phytol (2.82%) and glycerol 1-monolinolate (3.99%). The most dominant compound, an essential fatty acid was identified as linoleic acid (75.00%). Forty six compounds were identified in volatile oil (VO) extracted from NS seed oil by steam hydrodistillation (Table 2). VO extract consisted of a number of classes of compounds, were various types of nonterpenoid and terpenoid hydrocarbones. The major representative compounds of VO were p-cymene (3.81%), p-tert-butylpyrocatechol (4.28%), (+)-trans, trans-5-caranol (7.83%), 4-terpineol (10.55%), thymoquinone (13.53%) and thymol (31.98%). Thymol represented highest quantity. Other pharmacological minor compounds identified in VO were isothymol (also called carvacrol), carvone, copaene and limonene oxide. Chromatograms of ME and VO test fractions were given as Additional file 1: Data S1 and Additional file 2: Data S2, respectively.

**Table 1:** **Chemical composition of methanolic extract (ME) from *Nigella sativa *seed oils**

**Peak no.**	**Compounds**	**Retention time**	**Area%**
1	Limonene	11.138	1.13
2	Dihydrocarveol	13.709	0.08
3	4-Carvomenthenol	15.445	0.03
4	Thymoquinone	17.529	2.64
5	Isothymol	18.828	0.34
6	Verbenone	20.507	0.15
7	Humulen-(v1)	21.989	0.23
8	Butylated hydroxytoluene	24.380	2.44
9	Acetovanillone	25.395	0.94
10	3-Methoxy-4,5,6-trimethylphenol	28.170	0.05
11	4-(Methylthio)acetophenone	28.265	0.03
12	Myristic acid	29.861	0.06
13	Palmitic acid, methyl ester	33.175	0.10
14	(Z)6-Pentadecen-1-ol	34.024	0.53
15	Palmitic acid	34.248	2.77
16	Citronellyl butyrate	34.376	1.05
17	Oleic acid, methyl ester	36.567	0.49
18	Phytol	36.747	2.82
19	Linoleic acid	38.435	75.00
20	beta-Monolinolein	42.187	0.45
21	Benzedrex	42.672	0.35
22	Linoleoyl chloride	42.968	0.58
23	Silane, [1-(5-ethenyltetrahydro-5-methyl-2-furanyl)-1-methylethoxy] trimethyl-, trans-	45.655	1.74
24	Linoleic acid trimethylsilyl esterI	46.040	1.15
25	Glycerol 1-monolinolate	46.443	3.99
26	Acetylhydroquinone	51.604	0.14
27	Squalene	53.043	0.23
28	Stigmasterol	55.298	0.11
29	gamma-Sitosterol	56.448	0.21
30	Olealdehyde	58.703	0.17
			100.00

**Table 2:** **Chemical composition of volatile oil (VO) from *Nigella sativa *seed oils**

**Peak no.**	**Compounds**	**Retention time**	**Area%**
1	p-Cymene	11.008	3.81
2	beta.-Terpineol	12.233	0.48
3	Dihydrocarveol	13.044	0.50
4	Isothujol	13.144	0.61
5	(+)-trans, trans-5-Caranol	13.744	7.83
6	1-Terpinenol	13.818	0.23
7	cis-beta-Terpineol	14.332	0.17
8	Thujol	15.029	0.14
9	4-Terpineol	15.507	10.55
10	p-Cymen-8-ol	15.639	1.62
11	Terpineol	15.830	0.38
12	Allethrolone	16.156	0.21
13	Methoxycitronellal	16.615	0.12
14	Carvone	17.344	0.26
15	Thymoquinone	17.535	13.53
16	trans-Carveol	17.646	0.53
17	Linalyl anthranilate	17.766	0.27
18	Carvenone	17.858	0.14
19	Pichtosin	18.476	0.79
20	Thymol	18.565	31.98
21	Isothymol	18.961	1.64
22	trans,trans-2,4-Decadienal	19.257	0.31
23	Copaene	20.445	1.03
24	Limonene oxide	20.892	0.34
25	(+)-Sativen	21.892	0.12
26	Longifolene-(V4)	22.027	7.23
27	Methyl 2,5-octadecadiynoate	23.602	0.14
28	2-Tridecanone	23.750	0.63
29	beta.-Farnesene	24.249	0.16
30	(-)-Isoledene	24.832	0.16
31	p-tert-Butylpyrocatechol	25.421	4.28
32	beta-Pinene oxide	26.564	0.21
33	1-Cyclododecyl-ethanone	28.064	0.32
34	11-Dodecen-2-one	28.486	0.33
35	Epiglobulol	28.602	0.72
36	5-Tetradecen-1-ol, acetate, (Z)-	29.969	0.14
37	2,4,5-Trimethoxy benzoic acid	30.390	0.96
38	Palmitic acid, methyl ester	33.191	0.40
39	9-Octadecyne	34.033	0.56
40	Rimuen	34.317	1.31
41	Cephrol	34.378	1.24
42	Preg-4-en-3-one, 12,17-dihydroxy-20-nitrilo-	35.197	0.47
43	Methyl linoleate	36.474	0.97
44	Methyl oleate	36.564	0.37
45	Citronellal	36.700	1.70
46	Vellerdiol	37.558	0.10
			100.00

### Body weight, food consumption and weight of liver

The average body weights of normolipidemic rats were increased from 223 g to 279 g after 30-days duration. The body weight of HLP-C group was increased by 20% when compared to NLP-C rats after giving atherogenic suspension for 30 days. In treated groups with ME and VO for 30-days duration, the final body weights were significantly reduced by 28% and 25%, respectively after comparing to HLP-C control group. In the case of the average diet consumption in the all control and treated groups, there were no significant changes observed (data not shown). Average liver weights in HLP-C rats, were increased by 20% when compared to normolipidemic rats, while in the both treated groups, the liver weights were 5.48 and 5.43 g with highly significant reduction (Table 3).

**Table 3:** Average body and liver weights of rats during 30 days of treatment with ME and VO fractions

**Group**	**Body weight per rat (g)**		**Liver weight per rat (g)**
	**Prior to treatment**	**Post treatment**	
NLP-C	233.30 ± 20.8	278.60 ± 16.72	5.44 ± 0.22
HLP-C	22.00 ± 14.71	334.50 ± 14.50 (+20.06%)^α^	6.51 ± 0.01 (+19.65%)^α^
HLP- ME	221.25 ± 13.14	240.25 ± 9.74 (-28.18%)^b^	5.48 ± 0.03 (-15.82%)^b^
HLP-VO	220.00 ± 10.80	250.75 ± 4.85 (-25.04%)^b^	5.43 ± 0.07 (-16.59%)^b^

Each value is mean ± SD from 4 rats in each group except NLP (n = 5). NLP-C, normolipidemic control fed 1 ml saline/rat/day; HLP-C, hyperlipidemic control given 1 ml of saline before the feeding of 1 ml suspension containing 5 mg cholesterol, 30 mg coconut oil and 2.5 mg cholic acid /rat/day; whereas rats in HLP-ME, HLP-VO were fed 1 ml of 100 mg ME, 20 mg VO prior to administration of 1 ml of the above atherogenic suspension /rat/day for 30 days. Note: All the values in NLP-C and HLP-C control groups in this manuscript were already presented in our previously published article [37]. The values were statistically significant from NLP-C at ^*a*^*p* < 0.05 and HLP-C at ^*b*^*p* < 0.05.

### Impact on plasma and lipoprotein lipids of rats

**Figure 1:**
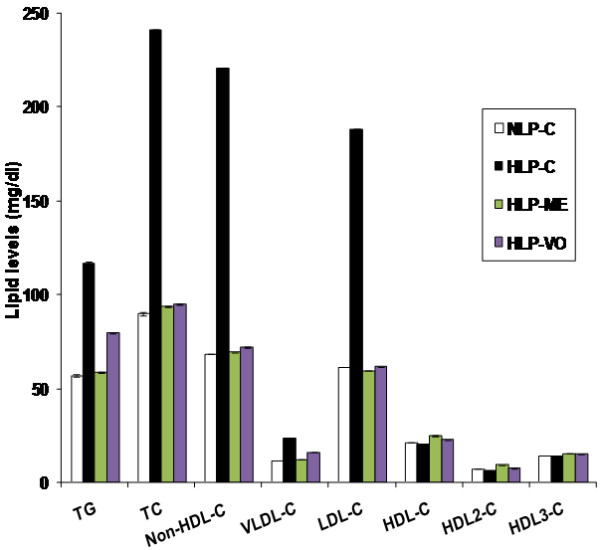
**Protective effects of ME and VO extracts of NS oil on plasma TG, TC, non-HDL-C (TC-HDL-C), VLD-C, LDL-C, HDL-C, HDL**_**2**_**-C and HDL**_**3**_**-C subfractions.** NLP-C, normolipidemic control fed 1 ml saline/rat/day; HLP-C, hyperlipidemic control given 1 ml of saline before the feeding of 1 ml suspension containing 5 mg cholesterol, 30 mg coconut oil and 2.5 mg cholic acid/rat/day; whereas rats in HLP-ME and HLP-VO were fed 1 ml of 100 mg ME or 20 mg VO, prior to administration of 1 ml of the above atherogenic suspension/rat/day for 30 days. Each value represents mean ± SD from pooled plasma samples in each group. The values of HLP-C control groups were statistically significant from NLP-C groups (*p* < 0.05), the values of treated groups were significantly different from HLP-C groups (*p* < 0.05), and the values between the treated groups were also significant (*p* < 0.05).

Figure 1 demonstrates that administration of atherogenic suspension to rats resulted in significant increments by 105% (TG), 168% (TC), 222% (non-HDL-C, equivalent to TC minus HDL-C), 105% (VLDL-C) and 207% (LDL-C) with substantial decrease by 6% (HDL-C) and 13% (HDL_2_-C), when compared to the values of normolipidemic rats. Pretreatment with 100 mg ME or 20 mg VO per rat per day for 30 days in atherogenic suspension fed rats, resulted in significant changes in TG (-50% or -32%), TC (-60% or -61%), non-HDL-C (-69% or -67%), VLDL-C (-49% or -32%), LDL-C (-68% or -67%) and HDL-C (+23% or +12%), HDL_2_-C (+51% or +21%) and HDL_3_-C (+11% or +8%), respectively, when compared to the corresponding values in HLP-C rats.

### Impact on HDL-linked arylesterase activity and LDL-C:arylesterase ratio

The data presented in Table 4, was basically obtained from Figure 1. In the lipidemic oxidative stress HLP-C control group, the value of arylesterase was significantly decreased by 21% coupled with increase in LDL-C:arylesterase ratio by 287% when compared to values in NLP-C rats. After treatment with the test fractions ME or VO, substantial restorations in arylesterase activity, and LDL-C:arylesterase ratio in the range of 102%-111%, were obtained.

**Table 4:** Effect of methanolic extract (ME) and volatile oil (VO) fractions on plasma arylesterase activity and the ratio of LDL-C and arylesterase activity in rats after 30 days of treatment

**Group**	**Arylesterase activity (nmole/min/ml)**	**LDL-C**^**†**^**:arylesterase activity**
NLP-C	665.15 ± 4.60	0.9220 × 10^-3^ ± 0.180 × 10^-5^
HLP-C	527.34 ± 4.73 (-20.71%)^a^	3.5682 × 10^-3^ ± 0.225 × 10^-5^ (+287.00%)^a^
HLP- ME	711.61 ± 4.70 (+34.94%)^b^	0.8338 × 10^-3^ ± 0.170× 10^-5^ (-76.63%)^b^
HLP-VO	680.57 ± 5.20 (+29.05%)^b^	0.9020 × 10^-3^ ± 0.180 × 10^-5^ (-74.72%)^b^

^†^LDL-C (mg/ml) values are taken from Figure 1. Each value represents mean ± SD from pooled plasma samples in each group. The values were statistically significant from NLP-C at ^*a*^*p* < 0.05 and HLP-C at ^*b*^*p* < 0.05, and the values between the treated groups were also significant (*p* < 0.05).

### Impact on hepatic 3-hydroxy, 3-methyl glutaryl CoA reductase activity

A rate limiting enzyme, hepatic 3-hydroxy, 3-methyl glutaryl CoA reductase (HMG-CoA reductase) activity was significantly reduced by 32% in HLP-C group, when compared to NLP-C value. Further, decline in the values of treated rats with ME or VO was observed by 44% or 42%, respectively, in comparison to NLP-C value (Table 5).

**Table 5:** ***In vivo *modulation effect of ME and VO fractions on hepatic HMG-CoA reductase activity in atherogenic suspension fed rats**

**Group**	**HMG-CoA reductase activity†**
NLP-C	28.90 ± 0.04
HLP-C	19.72 ± 0.07 (± 31.76%)^α^
HLP- ME	16.33 ± 0.06 (-43.49%)^a^
HLP-VO	16.74 ± 0.05 (-42.07%)^a^

† Its activity is expressed as percent of nmoles of HMG-CoA to mevalonate formed per mg liver protein. Each value represents mean ± SD from pooled liver samples in each group. The values were highly significant from NLP-C at ^*a*^*p* < 0.001, and the values between the treated groups were also statistically significant (*p* < 0.05).

### Impact on *ex vivo* and *in vitro* Cu^2+^-mediated oxidation of LDL and its subfractions sd-LDL and lb-LDL and their lag time

**Figure 2:**
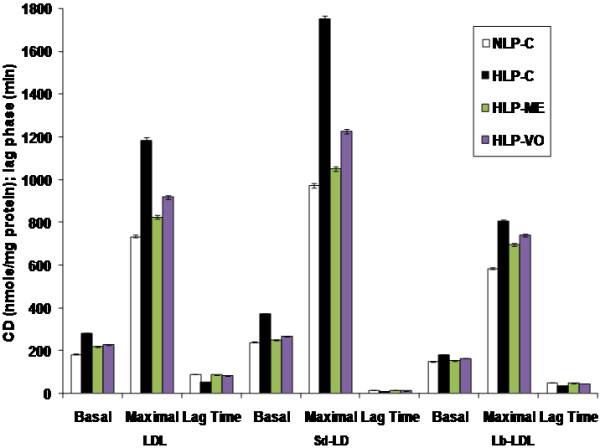
***In vivo *and *in vitro *Cu**^**2+**^**-catalyzed oxidation of LDL and its sd-LDL and lb-LDL subfractions from plasma of hyperlipidemic rats after 30 days of ME and VO treatment.** Here, expression of the conjugated diene (CD) values is as nmole malondialdehyde (MDA) equivalents/mg protein. The basal CD values show the status of oxidized LDL, and its subfractions sd-LDL and lb-LDL *in vivo*. After 2 h of incubation for lipoproteins, LDL, lb-LDL, and 30 min of incubation for sd-LDL with CuSO4, their maximal CD formation values were determined. The interval between the intercept of the tangent of the slope of the curve with the time in min is known as lag time. Each value represents mean ± SD from pooled plasma samples in each group. The values of HLP-C control groups were statistically significant from NLP-C groups (*p* < 0.05), the values of treated groups, HLP-ME and HLP-VO were significantly different from HLP-C groups (*p* < 0.05), and the values between the treated groups were also significant (*p* < 0.05).

As data in Figure 2 demonstrate that maximal conjugated diene (CD) formation from LDL was significantly increased by 299% in comparison to basal value in NLP-C rats. Feeding of atherogenic suspension to HLP-C rats led to significant increase in *ex vivo* basal CD formation of LDL, sd-LDL and lb-LDL by 52%, 55% and 20%, respectively in comparison to NLP-C rats. After treatment with ME or VO, significant reduction in basal CD formation of LDL and its subfractions, sd-LDL and lb-LDL, was seen by -22% or -18%, -32% or -28% and -14% or -8% when compared to the corresponding values in HLP-C rats. In comparison to basal CD values of LDL, sd-LDL and lb-LDL in NLP-C, the respective maximal CD values were increased by 299%, 305% and 290%. After treatment with ME or VO, a substantial decrease in CD product of LDL, sd-LDL and lb-LDL by 32% or 22%, 40% or 30% and 13% or 8%, respectively was observed when compared to atherogenic suspension fed control rats. In the case of lag phase of LDL, sd-LDL and lb-LDL lipoproteins, a substantial decrease by 41% (53 min), 35% (11 min) and 29% (36) in the stressed control group of rats was seen, when compared to the normolipidemic control rats. Similar to CD values, lag phase of the treated groups was significantly restored and increased close to the corresponding normal values in NLP-C rats (Figure 2).

### Impact on Cu^2+^-induced generation of malondialdehyde in plasma LDL, sd-LDL and lb-LDL

As in Table 6, basal malondialdehyde (MDA) formation of LDL, sd-LDL and lb-LDL was substantially increased by 106% (6.47 nmole/mg protein), 71% (8.05 nmole/mg protein) and 61% (6.18 nmole/mg protein), respectively in HLP-C rats when compared to the corresponding values in NLP-C rats after 30-days duration. In comparison to MDA values of HLP-C rats, the maximal MDA values of LDL, sd-LDL and lb-LDL were significantly increased by 81%, 66% and 32%, respectively. After treatment with the test fractions, ME or VO a significant basal restoration close to normal values was observed as 78% or 65%, 81% or 68%, and 80% or 72% and maximal restoration as 83% or 72%, 84% or 70% and 93% or 87%, respectively.

**Table 6:** ***In vivo *and *in vitro *Cu**^**2+**^**-induced generation of malondialdehyde (MDA) in plasma LDL, sd-LDL and lb-LDL from rats after 30 days of methanolic extract (ME) and volatile oil (VO) treatment**

**Group**	**Malondialdehyde**^**†**^					
	**LDL**		**Sd-LDL**		**Lb-LDL**	
	**Basal**	**Maximal**	**Basal**	**Maximal**	**Basal**	**Maximal**
NLP-C	3.14 ± 0.05	26.69 ± 0.11 (+750.00%)	4.71 ± 0.02	33.02 ± 0.09 (+601.06%)	3.83 ± 0.08	24.73 ± 0.05 (+549.69%)
HLP-C	6.47 ± 0.04 (+106.05%)^a^	48.40 ± 0.09 (+81.31%)^a^	8.05 ± 0.06 (+70.91%)^a^	55.60 ± 0.05 (+66.38%)^a^	6.18 ± 0.07 (+61.35%)^a^	32.68 ± 0.06 (+32.14%)^a^
HLP- ME	4.05 ± 0.08 (-37.40%)^b^	32.02 ± 0.06 (-33.84%)^b^	5.78 ± 0.05 (-28.19%)^b^	39.37 ± 0.06 (-29.19%)^b^	4.79 ± 0.09 (-22.49%)^b^	26.73 ± 0.07 (-18.20%)^b^
HLP-VO	4.80 ± 0.06 (-25.81%)^b^	36.98 ± 0.03 (-23.59%)^b^	6.92 ± 0.05 (-14.03%)^b^	47.08 ± 0.07 (-15.32%)^b^	5.30 ± 0.04 (-14.23%)^b^	28.30 ± 0.05 (-13.40%)^b^

^†^The values are expressed as nmole/mg protein. The basal MDA values in the table show the *in vivo* status of oxidized LDL, and its subfractions sd-LDL or lb-LDL. In the case of maximal MDA values of the lipoproteins (LDL and lb-LDL oxidation) were achieved after 2 h of incubation with CuSO_4_, whereas for sd-LDL maximal values were obtained after 30 min. Each value represents mean ± SD from LDL, sd-LDL and lb-LDL subfractions, isolated from pooled plasma samples in each group. The values were statistically significant from NLP-C at ^*a*^*p* < 0.05 and HLP-C at ^*b*^*p* < 0.05, and the values between the treated groups were also significant (*p* < 0.05).

## Discussion

Methanolic extract (ME) and volatile oil (VO) extracted from NS seed oil containing different phenolic, and terpenoid compounds along with other pharmaceutical compounds exert their effects on hypercholesterolemic rats through multiple mechanisms. In this study, we investigated hypolipidemic and antioxidant actions of ME and VO in atherogenic suspension fed rats. Administration of atherogenic suspension to rats containing 30 mg coconut oil, 5 mg cholesterol and 2.5 mg cholic acid per day/rat for 30 days exerted significant increments in the body and liver weights in HLP-C group of rats. Such gains in the weights were apparently owing to the deposition of lipid contents in the body, especially in the liver. Feeding of cholic acid, a component of atherogenic suspension helped in the intestinal absorption of the both cholesterol and fatty acids. This could be one of the reasonable factors that could explain the weight gains in the average body as well as the liver of the rats. Thirty day feeding of ME and VO suspensions significantly reduced the body weight along with liver weight, possibly by reducing the accumulation of fatty acids in adipose tissue of the treated rats, when compared to normolipidemic rats (Table 3).

As seen in HDL-C rats (Figure 1), increases in plasma TG and VLDL-C concentrations may be due to high production of VLDL with decreased clearance rate of TG as reported in hepatic β-VLDL secretion in cholesterol fed rabbits [46]. Presence of high concentration of plasma VLDL-C it may also lead to decreased production of antiatherogenic HDL because of the low availability of phospholipid remnants for its formation from VLDL and decline in the activity of lecithin cholesterol:acyltransferase as reported in several published reports. Treatment with the test fractions to the rats led to significant reduction in the levels of plasma TG, TC, VLDL-C, LDL-C and non-HDL-C, and antiatherogenic HDL-C along with its subfractions were significantly increased, and finally restoration of these altered lipoproteins were gained to near or above normal values. In this observation of results, ME had proven itself as higher potential agent than VO. Arylesterase is an important plasma HDL-linked antioxidant enzyme, protecting LDL from oxidative modification. LDL-C:arylesterase ratio, a good marker is used in hyperlipidemia. Thus, it is clear from the discussion that in lipidemic oxidative stress arylesterase, and LDL-C:arylesterase ratio were substantially changed apparently due to prooxidant effect against oxidation of LDL and ultimately malondialdehyde (MDA/TBARS) production. It is also evident from the discussion; decrease in arylesterase was consistent with HDL-C decrease and vice versa. Treatment with ME or VO to rats, restoration of arylesterase and HDL-C along with its subfractions was higher to normal values, and therefore arylesterase may play an important role in the antioxidation, anti-inflamatory or antiatherosclerotic properties [47].

An important rate-limiting enzyme, HMG-CoA reductase was suppressed in HLP-C rats by feeding atherogenic suspension (Table 5). Activity of HMG-CoA reductase in HLP-ME and HLP-VO rats was further reduced that apparently involved two related mechanisms as reported by Al-Naqeep *et al.*[48], in thymoquinone rich fraction treated rats: suppression of hepatic HMG-CoA reductase mRNA expression and increment in LDL receptor gene. Such regulations could clearly explain the hypolipidemic properties of ME and VO test factions that contained thymoquinone (2.64%) and (13.53%), respectively. Other compounds present in these test fractions may exert independently or synergistically (Tables 1 and 2) on lipidemic oxidative stress in rats. Atherogenic suspension induced oxidative stress and increased susceptibility of lipoproteins and other membrane lipids to oxidation in rats. Oxidative stress was result from equilibrium loss between antioxidant and oxidant systems. As in Figure 1 and Table 4, it is clearly evident that antioxidant HDL-linked arylesterase was significantly decreased and increased cholesterol content of LDL and LDL-C:arylesterase ratio in HLP-C rats compared to NLP-C rats. Increased LDL-C, low HDL-C and elevated TG levels (*lipid triad*) coupled with decrease in HDL-linked arylesterase in lipidemic oxidative stress rats are reflected in CD and MDA productions in LDL and its subfractions sd-LDL and lb-LDL. Sd-LDL subfraction of LDL is assumed comparatively higher inducer than lb-LDL for proatherogenic process in the vessel wall of humans with hyperlipidemia, similar to atherogenic lipoprotein phenotype pattern B subjects [49,50]. Because of several oxidizable properties were attributed to it, such as reduced content of free cholesterol and antioxidant, increased amount of more oxidizable polyunsaturated fatty acids [50,51]. Thus, more conjugated diene (CD) formation in sd-LDL than lb-LDL was seen in the both basal and maximal CD productions in lipidemic induced hyperlipidemic rats. Further, small lag time of sd-LDL of HLP-C rats compared to respective values in lb-LDL shows higher prone to oxidative damage. This could also reflect in increased Cu^2+^-induced *ex vivo* basal and maximal MDA production.

Administration of lipidemic stress in rats with the test fractions, ME and VO resulted in the restoration of CD and MDA levels of LDL and its subspecies sd-LDL and lb-LDL to an average value of 85%, when compared to the respective values in normolipidemic control rats, apparently through radical scavenging properties of the test factions [14,18]. Overall, hypolipidemic and antioxidant properties of ME were greater than VO in these stressed rats. Such effects of the test fractions are deciphered to indicate that the ME and VO strongly inhibit peroxidation of the molecules or resulted in elimination of the reactive toxic intermediates initiated by free radicals, thus preventing or delaying the cell damage. The above antioxidant and hypolipidemic actions of these test fractions are strongly supported by our previously published reports in which thymoquinone and limonene showed such strong effects [19,37]. However, the combined data from *in vivo* and *in vitro* studies strongly suggest that overall alleviation of ROS related disorders is due to highly effective amelioration of elevated levels of plasma atherogenic TG/TG rich lipoproteins, highly oxidizable and proatherogenic sd-LDL subspecies of LDL and low levels of atheroprotective HDL-C, i.e., *lipid triad*, coupled with a strong inhibition of *in vivo/ex vivo* and *in vitro* lipid peroxidation through efficient free radical scavenging properties of the test fractions and compounds in the order ME extract > pure thymoquinone > VO extract > pure limonene.

The above discussion conferred that the principal active constituents thymoquinone, limonene were identified as antioxidant as well as hypolipidemic agents along with linoleic acid (ω-6 fatty acid) that shows hypocholesterolemic activity in the presence of saturated fatty acid, palmitic acid in the ratio of 27:1, respectively. Thus, a combination of thymoquinone, limonene and linoleic acid in association with plamitic acid may be called as *compound triad* is highly effective in lipidemic oxidative stress when treated with ME. VO represented three related active compounds namely, thymol, thymoquinone and isothymol as antioxidants, they may be termed as *thyme triad*. VO, despite of being quite effective in alleviation of hyperlipidemia it lacks essential fatty acid and reduced form of limonene is comparatively less active than ME. It is apparently due to that limonene itself as hypolipidemic, and linoleic acid in the presence of palmitic acid show additive effects of thymoquinone for removal of lipid by suppression of HMG-CoA reductase activity and increased levels of LDL receptor in agreement with Al-Naqeep *et al.*[48] consequently resulted in significant decrease in oxidative stress in ME treated rats. Thus, it is clear that most abundant compound, linoleic acid in ME (Table 1) has a significant role in controlling the lipid levels and oxidative stress than VO. Because of VO, it showed lack of reduced form of limonene and absence of linoleic acid. However, the both test fractions likely share common mechanisms by suppressing HMG-CoA reductase activity, increased levels of LDL receptor [48], and free radical scavenging activity, other active constituents show independent or synergistic effects to alleviate lipidemic oxidative stress.

## Conclusions

The test fractions, ME and VO having thymoquinone common to the both extracts, and other pharmaceutical important compounds effectively ameliorated/normalized all the CVD risk parameters through modulation of HMG-CoA reductase activity, levels of LDL receptor and antioxidant mechanisms in atherogenic suspension fed rats. Thus, ME and VO both can be used as hypolipidemic, antioxidant, antiperoxidative and antiatherogenic agents in CVD and its complications. In future, it will need to elucidate the mechanisms of the principal effective compounds and their additive role of different combinations of the known compounds by GC-MS analysis of ME and VO to exert their lipid lowering and antioxidant actions, including the vascular status such as histopathological and plaque load studies after thorough evaluation study of toxic effect.

## Abbreviations

CD: Conjugated diene; CHD: Coronary heart disease; CVD: Cardiovascular disease; GC-MS: Gas chromatography–mass spectrometry; HDL-C: High density lipoprotein cholesterol; HLP-C: Hyperlipidemic control; HLP-ME: Hyperlipidemic ME; HLP-VO: Hyperlipidemic VO; HMG-CoA: 3-Hydroxy-3-methylglutaryl coenzyme A; Lb-LDL: Large buoyant-LDL; LDL-C: Low density lipoprotein cholesterol; MDA: Malondialdehyde; NLP-C: Normolipidemic control; NS: *Nigella sativa*; ME: Methanolic extract; ROS: Reactive oxygen species; Sd-LDL: Small dense-LDL; TBARS: Thiobarbituric acid reactive substances; TC: Total cholesterol; TG: Triglycerides; VLDL-C: Very low density lipoprotein cholesterol; VO: Volatile oil.

## Competing interests

The authors declare that they have no competing interests.

## Authors’ contributions

SA carried out the experiments, prepared the Tables, Figures, performed the statistical analysis, and wrote the manuscript. ZHB participated in the interpretation of the data and writing the manuscript. Both authors read and approved the final manuscript.

## Supplementary Material

Additional file 1: Data S1 Chromatogram of methanolic extract (ME) obtained from *Nigella sativa* seed oil. The each corresponding peaks were identified as given in Table 1. Click here for file

Additional file 2: Data S2 Chromatogram of volatile oil (VO) extracted from *Nigella sativa* seed oil. The each corresponding peaks were identified as given in Table 2. Click here for file
