# Supplementary figures and images for: Elucidation of mechanisms of actions of thymoquinone-enriched methanolic and volatile oil extracts from Nigella sativa against cardiovascular risk parameters in experimental hyperlipidemia
Source: Lipids Health Dis. 2013 Jun 13;12:86. doi: 10.1186/1476-511X-12-86 (PMC3693939; doi:10.1186/1476-511X-12-86)

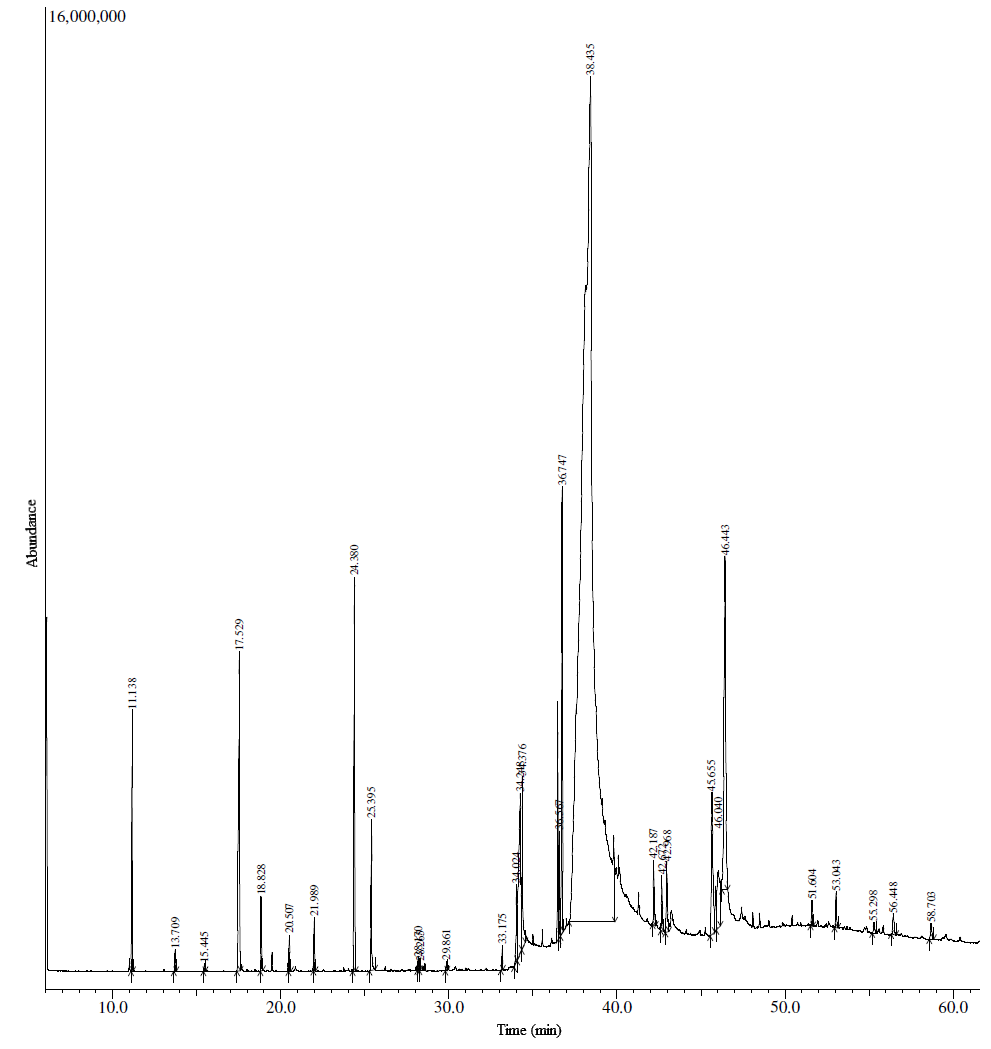

Supplement: Additional file 1: Data S1 — Chromatogram of methanolic extract (ME) obtained from Nigella sativa seed oil. The each corresponding peaks were identified as given in Table 1. [file 1476-511X-12-86-S1.tiff]

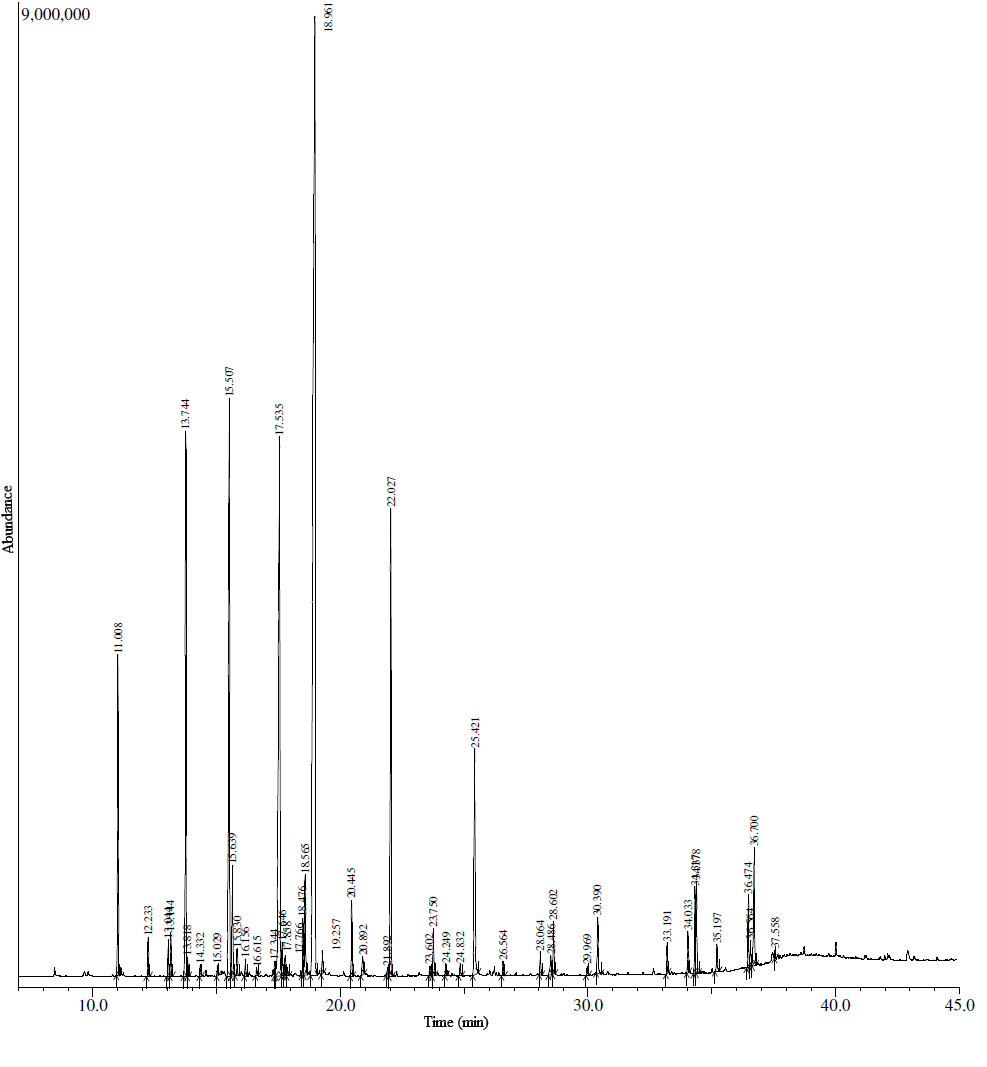

Supplement: Additional file 2: Data S2 — Chromatogram of volatile oil (VO) extracted from Nigella sativa seed oil. The each corresponding peaks were identified as given in Table 2. [file 1476-511X-12-86-S2.tiff]
